# Rapid publication-ready MS-Word tables for one-way ANOVA

**DOI:** 10.1186/2193-1801-3-474

**Published:** 2014-08-27

**Authors:** Houssein I Assaad, Lan Zhou, Raymond J Carroll, Guoyao Wu

**Affiliations:** Department of Statistics, Texas A&M University, College Station, TX 77843 USA; Department of Animal Science, Texas A&M University, College Station, TX 77843 USA; Department of Medical Physiology, Texas A&M University, College Station, TX 77843 USA

**Keywords:** Statistical analysis, Multiple comparisons, Online software, Computation, Biology, R, Shiny

## Abstract

**Background:**

Statistical tables are an important component of data analysis and reports in biological sciences. However, the traditional manual processes for computation and presentation of statistically significant results using a letter-based algorithm are tedious and prone to errors.

**Results:**

Based on the R language, we present two web-based software for individual and summary data, freely available online, at http://shiny.stat.tamu.edu:3838/hassaad/Table_report1/ and http://shiny.stat.tamu.edu:3838/hassaad/SumAOV1/, respectively. The software are capable of rapidly generating publication-ready tables containing one-way analysis of variance (ANOVA) results. No download is required. Additionally, the software can perform multiple comparisons of means using the Duncan, Student-Newman-Keuls, Tukey Kramer, and Fisher’s least significant difference (LSD) tests. If the LSD test is selected, multiple methods (e.g., Bonferroni and Holm) are available for adjusting p-values. Using the software, the procedures of ANOVA can be completed within seconds using a web-browser, preferably Mozilla Firefox or Google Chrome, and a few mouse clicks. Furthermore, the software can handle one-way ANOVA for summary data (i.e. sample size, mean, and SD or SEM per treatment group) with post-hoc multiple comparisons among treatment means. To our awareness, none of the currently available commercial (e.g., SPSS and SAS) or open-source software (e.g., R and Python) can perform such a rapid task without advanced knowledge of the corresponding programming language.

**Conclusions:**

Our new and user-friendly software to perform statistical analysis and generate publication-ready MS-Word tables for one-way ANOVA are expected to facilitate research in agriculture, biomedicine, and other fields of life sciences.

## Introduction

Statistical tables are ubiquitous in agricultural, biological, and biomedical studies (Steel et al. 1997). An example is shown in Table 1, reporting the effects of oral administration of interferon tau (IFNT) on concentrations of amino acids, glucose, lipids, and hormones in the plasma of Zucker diabetic fatty (ZDF) rats (Tekwe et al. 2013). Here, we focus on generating tables from one-way analysis of variance (ANOVA) models where measurements are summarized as mean ± SEM for each treatment group. Typically, post-hoc test results are also included in these tables using a letter-based algorithm (Piepho 2004) to indicate which treatment groups are significantly different. With this algorithm, means for treatments are assigned letters (e.g., a, b, and c) to highlight significant differences. Those means that are not significantly different are assigned a common letter. In other words, two treatments without a common letter are statistically significant at the chosen level of significance (e.g., *P* ≤ 0.05 or ≤ 0.01). The Tukey-Kramer (TK), Student-Newman-Keuls (SNK), Fisher’s least significant difference (LSD), Duncan (DC), and Bonferroni (BF) tests are among the most popular multiple comparison procedures used in life science research (Steel et al. 1997), including amino acid biochemistry, nutrition, pharmacology, and physiology (Wang et al. 2014a, b ; Wu and Meininger 1997; Wu 1997).

**Table 1:** **Effects of oral administration of IFNT on concentrations of amino acids, glucose, lipids and hormones in the plasma of ZDF rats**

Metabolites or hormones in plasma	Oral IFNT dose (μg/kg BW/day)		
	0	4	8
Arginine, μM	110 ± 4^b^	115 ± 5^b^	149 ± 6^a^
Valine, μM	219 ± 9^a^	201 ± 7^a^	172 ± 6^b^
Isoleucine, μM	208 ± 8^a^	206 ± 7^a^	178 ± 6^b^
Leucine, μM	245 ± 10^a^	233 ± 9^a^	196 ± 8^b^
Glucose, mM	24.5 ± 0.3^a^	23.8 ± 0.4^a^	21.9 ± 0.4^b^
Free fatty acids, mM	1.60 ± 0.06^a^	1.53 ± 0.05^a^	1.34 ± 0.05^b^
Triacylglycerol, mM	6.05 ± 0.13^a^	5.90 ± 0.27^a^	5.17 ± 0.11^b^
Total cholesterol, mM	5.18 ± 0.23^a^	4.94 ± 0.24^a^	4.23 ± 0.13^b^
Insulin, pM	307 ± 10	294 ± 11	301 ± 7
Adiponectin, mg/L	2.78 ± 0.08	2.94 ± 0.09	2.64 ± 0.13
Leptin, μg/L	19.6 ± 1.2^b^	19.3 ± 1.0^b^	13.7 ± 0.9^a^

Adapted from Tekwe et al. (2013). Plasma samples were obtained from 12-week-old rats. Values are the means ± SEM, n = 6 per treatment. ^a-b^Means in a row without a common superscript letter differ (*P* < 0.05), as analyzed by one-way ANOVA.

In this paper, we introduce two software, freely available online, at (http://shiny.stat.tamu.edu:3838/hassaad/Table_report1/ and http://shiny.stat.tamu.edu:3838/hassaad/SumAOV1/) for one-way ANOVA. The software are capable, within few clicks, of generating publication-ready MS-Word tables corresponding to multiple data sets, and of exporting them to Microsoft Word or any RTF reader, with all the post-hoc tests results being included therein. The software can also handle situations where only summary data are available (i.e., sample size, mean, and SD or SEM per group), without the need to use the original individual observations. We believe that our new method will save biologists, and applied scientists in general, an ample amount of time and avoid inputting, by hand, superscript letters (see Table 1) derived from the appropriate statistical tests. This offers a distinct advantage over the traditional manual processes for computation and presentation of results in tables that are not only tedious but are also prone to errors.

Several software packages can perform one-way ANOVA, followed by post-hoc analysis (e.g., R, SAS, JMP, and SPSS). To our knowledge, none of them is capable of exporting the multiple comparison results into an RTF reader in a format similar to that of Table 1 without advanced knowledge of the corresponding programming language. Also, SAS, SPSS and JMP are not free. The main challenge lies in exporting the superscripts used to summarize the significance results to an RTF reader. A simple Google search of the terms “ANOVA calculator” or “ANOVA from summary data” reveals many free web-based programs^a^ that can construct ANOVA tables based either on original or summary data. Despite their simple interface, these programs suffer from major drawbacks. The majority cannot perform post-hoc analysis of any kind. Additionally, none of them can export results to an RTF reader in a publication-ready format, making their usage by a broad community very unlikely. To overcome these disadvantages, we wrote our software in the R language (R core Team, 2014) and used the following R packages: grifExtra (Auguie 2012), XLConnect (Mirai Solutions GmbH 2014), agricolae (Mendiburu 2014), rtf (Schaffer 2013), and shiny (Rstudio Inc 2013).

In the following sections, we introduce necessary background materials for one-way ANOVA coupled with multiple comparison techniques. The main goal is to highlight some of the limitations of the statistical tests included in the software. We also wanted to underline the necessary assumptions required by one-way ANOVA and emphasize that the software should be used only when such assumptions are nearly satisfied. In addition, we present several options to prepare the data for input into the software. Different toy datasets can be downloaded from the software webpage to be used throughout the paper to illustrate the functionality of our software. We also describe the different components of the software and the steps required to generate the tables in MS Word. Furthermore, we offer various tips and useful links to cover more input and output scenarios. Concluding remarks are given towards the end of this article.

## Background and materials

### 1. One-way ANOVA

Here, we present a brief non-technical description of one-way ANOVA and introduce few terms that will be used throughout the rest of this paper. One-way ANOVA, also known as single-factor ANOVA, involves the analysis of data sampled from two or more numerical populations (probability distributions). The characteristic that labels the different populations is called the *factor* under study. This factor variable can take different values known as *factor levels*. For example, in a published study involving dietary supplementation with 0, 0.5, 1, 2, and 4% monosodium glutamate to young pigs (Rezaei et al. 2013), the experiment consisted of one factor (i.e., monosodium glutamate) with five different levels. Also, let us consider an experiment to assess the effect of four brands of gasoline automobile on engine operating efficiency (measured in mpg). Here, the brand of gasoline is the factor variable and it has four levels (the four brands). The response variable is the engine operating efficiency. One-way ANOVA assumes that the numerical populations or probability distributions of each factor level follow a normal distribution with a common variance, and differ only with respect to their means. Therefore, differences in the means reflect the effect of the essential factor levels, and it is for this reason that ANOVA focuses on the mean responses for the different factor levels. If the factor has only two levels, ANOVA is equivalent to an unpaired t-test comparing two group means. One-way ANOVA usually proceeds in two steps. First, it determines whether or not the factor level means are the same using an overall test. Second, if the factor level means differ, the researcher will conduct a follow-up analysis, known-as *post-hoc analysis*, to examine how they differ. Our software offers a variety of statistical tests to perform pair-wise comparisons in the post-hoc analysis step.

### 2. Multiple comparison methods

The main purpose of this section is to provide the reader with some insight into the limitations of the different testing procedures available in the software. For any testing problem, there are two types of errors^b^. A *false positive* (also called *Type I error*) occurs when we detect an effect that does not really exist. A *false negative* (*Type II error*) occurs when we fail to declare a truly existing effect. Most of the classical multiple comparison procedures (MCP), such as the DC, SNK and LSD tests, control the Type I error [more precisely, the family-wise error rate (*FWER*), which is the probability of committing at least one Type I error in a series of hypotheses testing] in the *weak* sense. Namely, all computations (e.g. p-values) are conducted under the assumption that all null hypotheses are true. In practice, this assumption is rarely expected to hold, allowing the Type I error to be in excess of the usual 5% value. Therefore, a stronger control for Type I error rate under less restrictive assumptions is often required. A MCP controls the Type I error rate in the *strong* sense if this error is controlled under any partial configuration of true and false hypotheses. While TK and BF do control the FWER in the strong sense, they have a relatively low *power*. In other words, TK and BF are more likely to correctly identify true hypothesis as being true, but also might fail to declare false hypothesis as being false (the two methods generate larger p-values than they truly are). A summary of the previous discussion is given in Table 2, which is taken from Christensen ( 2011) with some modifications. Ideally, it is desired to choose a method that controls the FWER in the strong sense, while achieving the highest possible power. Increasing the power can be done by extending *single-step*^c^ testing procedures into *stepwise* procedures via a technique known as *the closure principle* (Bretz et al. 2010). For instance, the stepwise Holm procedure is an extension of the single-step BF test. By construction, step-wise procedures are more powerful and control the FWER in the strong sense. The general recommendation is to use a testing procedure that controls Type I error in the strong sense while accounting for *logical constraints*^d^ and potential correlation among the tests. The books by Westfall et al. ( 2011) and Bretz et al. ( 2010) offer a thorough and accessible introduction to the MCP. Furthermore, these books provide the necessary code in SAS and R, respectively.

**Table 2:** **Summary of multiple comparison methods**

LSD	Highest error rate and power of any method. In general, it controls the FWER in the weak sense; when there are 3 treatment groups, the FWER is controlled in the strong sense.
BF	Controls the FWER in the strong sense, but it is too conservative (reduces the number of true positives).
TK	Lowest error rate and power, control the FWER in the strong-sense
SNK	Error-rate and power intermediate between TK and DMRT. Controls the FWER in the weak sense.
DC	Error-rate and power intermediate between SNK and LSD. Controls the FWER in the weak sense.

The table was taken from Christensen ( 2011) with some modifications.

BF = Bonferroni; DC: Duncan method; LSD = Least significant difference; SNK = Student-Newman-Keuls; TK = Tukey Kramer (or Tukey HSD in balanced designs).

## The software

### 1. Working with software 1

**Figure 1:**
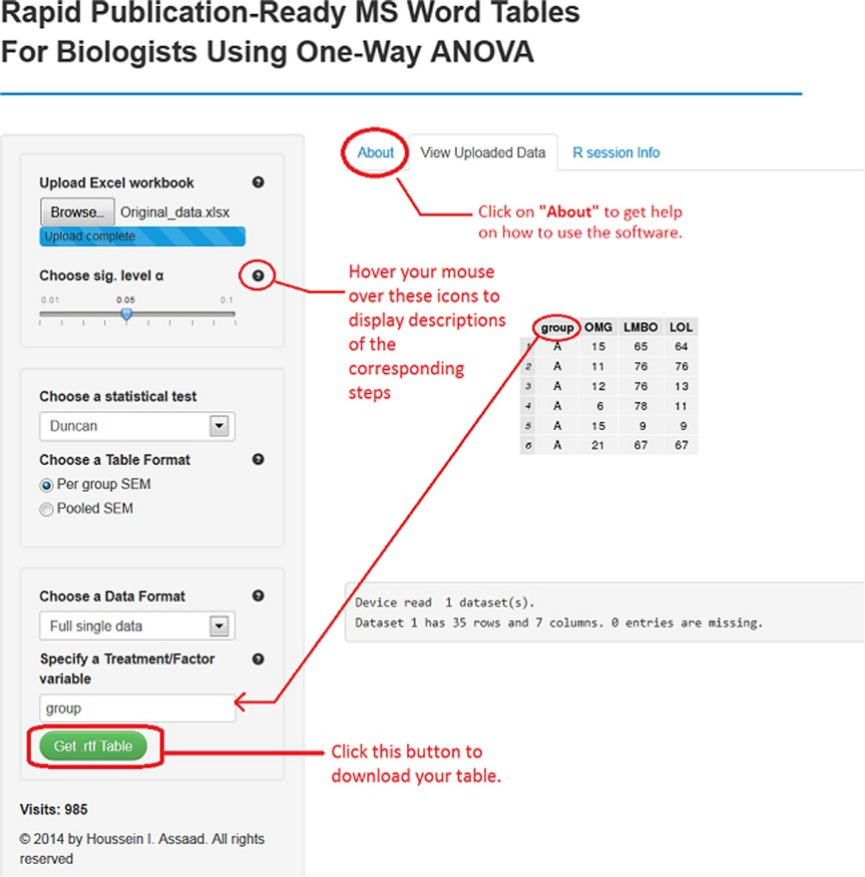
**A screenshot of software 1 for setting (S1).**

Software 1 (http://shiny.stat.tamu.edu:3838/hassaad/Table_report1/) can handle multiple scenarios where data should be arranged accordingly to obtain the sought results without generating errors. For illustration purposes, *different toy data sets that correspond to each scenario can be downloaded*^*e*^*from the software webpage under the “About” panel* (see Figure 1). We distinguish the following settings:

1. A single data set arranged in one Excel sheet: The file should be saved as an Excel workbook ‘Filename.xls’ or ‘Filename.xlsx’, depending on which version of Microsoft Excel the researcher is using (see file ‘Single_data.xlsx’).
2. Multiple data sets arranged within multiple Excel sheets (one data set per sheet) and saved in one Excel workbook (see file ‘Multiple_data.xlsx’).
3. Single data set of summary measurements arranged in one Excel sheet (see file ‘Single_Summary_Data.xlsx’).
4. Multiple data sets of summary measurements in multiple Excel sheets (see file ‘Multiple_Summary_Data.xlsx’).

For the first two scenarios, data rows should correspond to different subjects or experimental units, whereas data columns should describe different variables. The Excel sheets must only contain the data set without any comments or explanations (see file ‘Single_data.xlsx’ for example). Also, an appropriate name should be assigned to each variable. Note that each data set (in one Excel sheet) should contain exactly one factor variable and at least one response variable. For instance, the file Single_data.xlsx contains a single data set with one factor variable (group) with four levels A, B, C and D and six response variables V1 to V6. In this case, the software will conduct six one-way ANOVAs, one for each response variable, and summarize the results in one table in a format similar to Table 1. It should be borne in mind that all the six one-way ANOVAs share the same factor variable ‘group’. *Missing values should be left as empty cells*. The data set, within an Excel sheet, doesn’t have necessarily to start from the top left cell in Excel (cell A1), as long as the tabular (rectangular) form is maintained.

**Figure 2:**
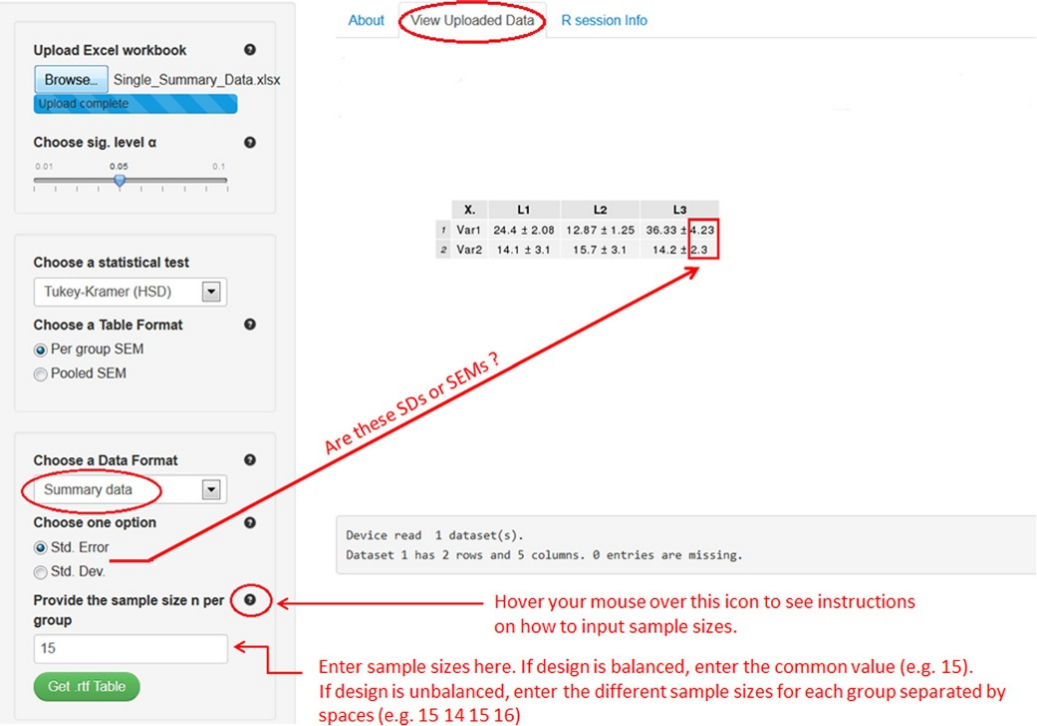
**A Screenshot of software 1 for setting (S3).**

The last two scenarios are especially useful in cases where the original individual observations are not available, and where only the sample size, mean, and SD or SEM for each factor level are known. For example, this might happen, if the researcher wants to analyze data that have been summarized in a submitted or published article. Refer to files Single_Summary_Data.xlsx and Multiple_Summary_Data.xlsx to prepare data for scenarios (S3) and (S4), respectively. Note that the software can acquire the mean and SEM or SD for each treatment group from the summarized table, but requires the user to enter the sample size. For example, consider the file Single_Summary_Data.xlsx, which has two response variables Var1 and Var2 and one factor variable with 4 levels L1 to L4. By uploading this file into the software, it will automatically detect the mean and SEM or SD for each group for all the response variables. All is left now is to specify the sample sizes as shown in Figure 2. If the design is balanced, enter the common value for sample size per group (e.g., 15). If the design is unbalanced, enter one value for each factor level, *in the order they appear in the Excel file*, separated by spaces (e.g., 15 14 15 16 for L1 to L4, respectively). Having borders around the researcher’s table cells does not affect the functionality of the software. In the next section, we present software 2 that offers a more user-friendly interface to deal with summary data. At first, it might seem that one should make some effort to get the summary data ready for the software in scenarios S3 and S4 (see Single_Summary_Data.xlsx). However, several free online programs^f^ are currently available to convert a PDF document, which is the standard format for submitted or published papers, to a Word file. Once the table is opened in Word, it can be copied to Excel and then loaded into the software *after removing all the superscripts from the table*. The latter procedure can be done easily using the “Find and Replace” feature (click CTRL + F to open it) in Excel by replacing all the superscripts with an empty space.

The user of our software should follow the steps below:

1. Upload an excel workbook (both .xls and .xlsx format are supported) and select the level of significance α.
2. Specify a statistical test to perform all pair-wise comparisons. Currently available are the Tukey-Kramer (also known as Tukey’s HSD) test, the Duncan test, the Student-Newman-Keuls (SNK) test, and the least significant difference (LSD) test. If the researcher selects the LSD test, multiple methods (e.g., Bonferroni and Holm) are available for adjusting p-values.
3. Choose the table’s output format. Two formats are widely used in the literature. By selecting ‘Per group SEM’, the table will report the mean and SEM for each group (see Table  1). The ‘Pooled SEM’ option will only report the means for each group and one pooled SEM for all the treatment groups (see Table  3).
4. Choose a data format. For setting (S1) select “Full single data” and then specify the factor variable in the researcher’s data set (names are case-sensitive). For (S2) select “Workbook (multiple sheets)”. Choose “Summary data” for setting (S3), while for (S4) select “Workbook (multiple sheets)” and check the summary data checkbox. For both (S3) and (S4), the researcher has to provide the sample size per group, as well as SDs or SEMs in the summary data.
5. Click on the green button to download the table with all statistical results included.

**Table 3:** **Effects of oral administration of IFNT on concentrations of amino acids, glucose, lipids and hormones in the plasma of ZDF rats**

Metabolites or hormones in plasma	Oral IFNT dose (μg/kg BW/day)			
	0	4	8	Pooled SEM
Arginine, μM	110^a^	115^a^	149^b^	5.0
Valine, μM	219^a^	201^a^	172^b^	6.2
Isoleucine, μM	208^a^	206^a^	178^b^	5.1
Leucine, μM	245^a^	233^a^	196^b^	7.0
Glucose, mM	24.5^a^	23.8^a^	21.9^b^	0.3
Free fatty acids, mM	1.6^a^	1.53^a^	1.34^b^	0.04
Triacylglycerol, mM	6.05^a^	5.9^a^	5.17^b^	0.14
Total cholesterol, mM	5.18^a^	4.94^a^	4.23^b^	0.15
Insulin, pM	307	294	301	5.3
Adiponectin, mg/L	2.78	2.94	2.64	0.06
Leptin, μg/L	19.6^a^	19.3^a^	13.7^b^	0.87

Adapted from Tekwe et al. (2013). Plasma samples were obtained from 12-week-old rats. Values are the means, n = 6 per treatment. ^a-b^Means in a row without a common superscript letter differ (*P* < 0.05), as analyzed by one-way ANOVA.

The publication-ready table for one-way ANOVA and multiple comparison results should now open in the MS Word or in the default RTF reader on the researcher’s computer system. The table can now be edited as desired (e.g., adding rows, columns, and borders).

### 2. Working with software 2

**Figure 3:**
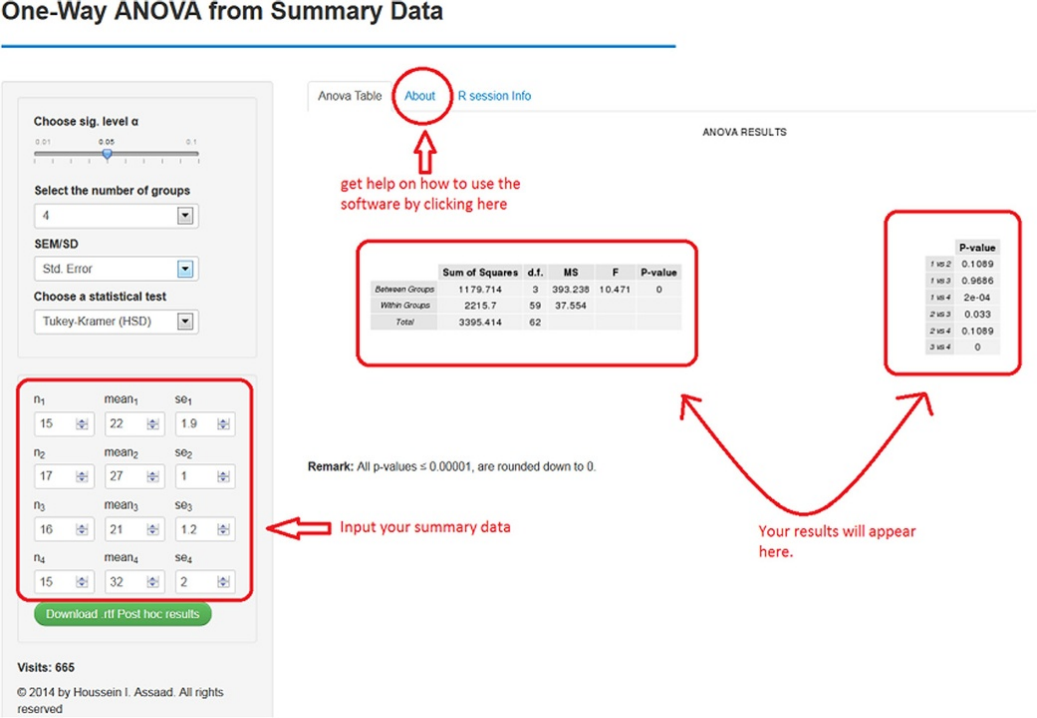
**A screenshot of software 2.**

Our main intention behind this software (http://shiny.stat.tamu.edu:3838/hassaad/SumAOV1/) is to provide reviewers of scientific papers with a quick and simple tool to check the statistical results summarized in a certain table. This method might be cumbersome if used to check results in a relatively large table containing several response variables (e.g., Table 1) because results must be checked one row at a time. An efficient alternative is to consider using software 1 under the (S3) and (S4) settings, which allow researchers to feed the whole table to the software at once *after removing the superscripts from it* (after all, the main goal is to check whether these superscripts are correct!). The user should carry out these steps in the given order (see Figure 3):

1. Choose the level of significance α. By default, it equals 0.05.
2. Indicate the number of treatment/group means to compare. Enough fields to input the researcher’s data will be available based on that number.
3. Specify whether the researcher will provide SD or SEM for each group.
4. Select a statistical test for pair-wise comparisons.
5. Input the
  - Sample size for each group, n_1_, n_2_, n_3_, etc.
  - Mean (average) for each treatment group, mean_1_, mean_2_, mean_3_, etc.
  - The SEM or SD for each treatment group, depending on the researcher’s selection in step 3.

The ANOVA table and a table containing all the pair-wise comparisons should now appear on the right (see Figure 2). *Note that the results will be automatically updated if the researcher introduces any changes to their input* (e.g., changing the statistical test and sample sizes).

### 3. Caution regarding the names of variables

Spaces in variable names should be avoided as they might lead the software to generate an error instead of a correct table output. Also, if the length of a variable’s name in the dataset is larger than 10 characters, which might be the rule rather than the exception in many cases in biological studies, the software will abbreviate the variable’s name. This can lead to ambiguous or unpleasant terms. We, therefore, advise researchers to subjectively assign descriptive abbreviations for variables with long names before loading their dataset into the software.

### 4. Transposing the output table

The software generates a table for one-way ANOVA and multiple comparisons in a format similar to that of Table 1. The response variables are in different rows and the factor levels occupy different columns. We do not include a functionality that reverses this order because such a task can be easily done in Word or Excel. Typing “Transposing table in Word/Excel” in the Google search engine return many helpful links. Choose the one that corresponds to the researcher’s version of Word or Excel.

## Concluding remarks

We presented two free web-based software capable of generating publication-ready RTF tables for one-way ANOVA with pair-wise comparison results included therein. These tables are often prepared for writing agricultural, biological, and medical science papers. Due to its simple interface, the software spare the researcher a considerable amount of time and eliminate errors introduced by human input. The software can handle an Excel workbook with multiple datasets saved in multiple sheets, creating one table per dataset. Our software also support two of the most commonly used table outputs in life science articles (see Tables 1 and 2 for example). Additionally, tables can be generated based solely on summary results (i.e., the sample size, mean, and SD or SEM for each treatment group). This need might arise if the researcher wants to analyze data that have been summarized in a submitted or published manuscript. The software can be extended in several directions. For instance, it is possible to include additional multiple comparison tests that might improve the power of the currently available methods. Another option is to cover more families of elements to be tested, in addition to all pair-wise comparisons, such as general contrasts and linear functions.

## Endnotes

^a^See http://statpages.org/anova1sm.html, http://vassarstats.net/anova1u.html, and http://www.danielsoper.com/statcalc3/calc.aspx?id=43.

^b^There is also a Type III error in two-sided test problems. It is defined as the correct rejection of the null hypothesis coupled with a wrong directional decision.

^c^When testing multiple hypotheses, a test procedure is called a *single-step* method if the rejection or non-rejection of a null hypothesis does not take the decision of any other hypothesis into account, e.g. the BF and TK tests. On the other hand, *step-wise* methods differ from single-step procedures in that the results of a given test depend upon the results of other tests, e.g., Holm.

^d^For example, consider all pair-wise comparisons of 3 treatment means M_1_, M_2_ and M_3_. If M_1_ ≠ M_2,_ then logically, M_1_ = M_3_ and M_2_ = M_3_ cannot be true simultaneously. Choosing a test that does not account for these *logical constraints* might lead to problems with the interpretation of the test results.

^e^Access to Dropbox is required in order to download the corresponding toy datasets.

^f^See http://www.pdfonline.com/pdf-to-word-converter/.

## Availability and requirements

- **Project name:** Rapid publication-ready MS Word tables for one-way ANOVA.
- **Project home page:**http://shiny.stat.tamu.edu:3838/hassaad/Table_report1/ and http://shiny.stat.tamu.edu:3838/hassaad/SumAOV1/.
- **Operating system(s):** Platform independent.
- **Programming language:** R, HTML/CSS, RTF.
- **Other requirements:** internet connection, Mozilla Firefox, or Google Chrome.
- **Any restriction to use by non-academics:** None.

## Abbreviations

ANOVA: Analysis of variance
IFNT: Interferon tau
SAS: Statistical analysis system
SD: Standard deviation
SEM: Standard error of the mean
ZDF: Zucker diabetic fatty.
